# Scalp Wounds

**Published:** 1900-03

**Authors:** W. E. Fowler

**Affiliations:** Huntsville, Texas


For Texas Medical Journal.

### Scalp Wounds.

BY W. E. FOWLER, M. D., HUNTSVILLE, TEXAS.

We are taught in the colleges we attend, in the works on surgery we read, and by the majority of the contributors to the current literature found in the medical journals, that scalp wounds are among the most intractable that we are called upon to treat, so far as union by first intention is concerned. We are taught that this class of wounds is prone to suppuration, followed by all the long trains of troublesome sequelæ of septic conditions. Why this is so I am unable to say, since the personal experience derived from my practice convinces me that these wounds are not more stubborn or annoying than those inflicted on other parts of the body, provided they are managed according to the technique hereafter to be described. But this assertion is subject to qualifications that seem to have evaded the notice of many medical writers after whom I have read. In the first place, the scalps of the majority of people are not, as a rule, kept as clean as many other portions of the body; and this being true, it should not seem strange that its wounds are more likely to be contaminated by the septic and deleterious particles clinging to their edges.

Some very successful surgeons advocate the plan of closing these wounds simply by using the hair instead of sutures; but if I saw one thus closed, I should be astonished if suppuration did not follow. Such a mode of procedure is inexcusable in this day of enlightened surgery—when even the most rustic practitioner is commonly found to be very well versed in the germ theory, and in the potency and use of antiseptics. Others, with long records of eminently successful operations, teach that it is better to close these wounds with strips of adhesive plaster. Here, again, I should expect bad results. In fact, I am opposed to the closure of any class of wounds with plaster, because it always furnishes a nidus with wide open mouth to receive and retain filth.

During the past year I have treated innumerable scalp wounds—most of them coming under the head of *contused,* having been produced by the use of some kind of "blunt instruments," as the newspapers generally put it; yet I have had no suppurations—all having healed by what is commonly called first intention. So, therefore, comparing my results with what I have been taught to expect, I have concluded to append my technique. As soon as the clotted blood is washed off, I shave away the hair with a keen razor. As this shaving process is sometimes objected to by the patient or his friends, I will state that it is not necessary to clear away the hair for more than a half inch on either side of the wound; and this should be explained to those who object. Having thus cleared the locality of a sufficient quantity of hair, I thoroughly cleanse the wound with warm carbolized water—being careful to remove all foreign bodies, such as dirt and other debris. I next approximate the lips of the wound and close with silk sutures; and these sutures are inserted and brought out at least a quarter of an inch from the lips of the wound, and I am always very careful that the needle should carry them to its floor.

The wound having thus been closed, I spray with listerine and seal with absorbent cotton, collodion and iodoform.

The collodion with which I close the wound is ten per cent. iodoform, and I apply it as follows: With a swab made by wrapping a piece of absorbent cotton around the point of a probe, or a clean stick, I brush the preparation over the wound and the clean surface surrounding it. Before this has time to dry, I put on a thin layer of cotton and immediately cover it with the solution. The cotton will readily become thoroughly saturated, and at the end of a few moments will be dry, leaving the wound completely sealed with a firm dressing which, save for looks, needs no bandage. On the fifth day I remove the dressing, take out the stitches and discharge the patient—cured.

Any bleeding points can be controlled—as a general rule—by torsion; but if not, then the usual method must be resorted to.

Some may ask why I insert my sutures so far away from the edges of the wound. My answer is this: While the scalp is very vascular, it is thick and firm; and, if the sutures are placed too near the edges, the force necessary for complete closure will almost invariably destroy the circulation within their radii and they will slough out. But, by the method I follow, less force is necessary in order to closure, thereby obviating the destruction of circulation. If the pericranium is cut, it should first be closed with a sublayer of catgut sutures.

The above plan of procedure is simple, and no one who follows it will be disappointed; and I would say, in closing, that interrupted sutures should be used in this class of wounds.

